# Optical Coherence Tomography Angiography Evolution of Choroidal Neovascular Membrane in Choroidal Rupture Managed by Intravitreal Bevacizumab

**DOI:** 10.1155/2019/5241573

**Published:** 2019-01-06

**Authors:** Massimo Lorusso, Luisa Micelli Ferrari, Eleni Nikolopoulou, Tommaso Micelli Ferrari

**Affiliations:** ^1^Department of Ophthalmology, Ente Ecclesiastico “F. Miulli” Hospital, Acquaviva delle Fonti, Bari, Italy; ^2^Department of Ophthalmology, “G. Moscati” Hospital, Taranto, Italy

## Abstract

**Purpose:**

To describe a case of a 25-year-old man with choroidal neovascularization (CNV) secondary to traumatic choroidal rupture treated with intravitreal bevacizumab and to evaluate the vascular structure of the area near the traumatic choroidal rupture.

**Methods:**

The patient underwent complete ophthalmologic evaluation, including best-corrected visual acuity (BCVA), intraocular pressure, anterior segment and funds examination, and optical coherence tomography angiography (OCTA) at baseline and on each follow-up visit. Fluorescein angiography (FA) was performed at baseline. Intravitreal bevacizumab was administered at the time of choroidal neovascular membrane diagnosis.

**Results:**

At baseline, ophthalmoscopic examination of the left eye revealed four subretinal macular hemorrhages and two choroidal ruptures located temporally to the fovea. On OCT angiograms, the choroidal rupture appeared as a hypointense break in choriocapillaris plexus. At 4-week follow-up, the OCTA disclosed a well circumscribed lesion characterized by numerous and fine anastomotic vessels. Patient received intravitreal injection of bevacizumab. At 6-week post injection, OCTA documented regression of the neovascular complex.

**Conclusion:**

Choroidal neovascularization is a common complication associated with traumatic choroidal rupture and OCTA may represent a complementary diagnostic technique to evaluate the vascular structure of the area near the traumatic choroidal rupture.

## 1. Introduction

Choroidal rupture is a break in the choroid, Bruch membrane, retinal pigment epithelium (RPE) that results from blunt ocular trauma. In most cases choroidal ruptures are secondary to indirect trauma, non-penetrating closed-globe blunt trauma [1]. After blunt trauma, the ocular globe undergoes mechanical compression and then sudden hyperextension. The sclera and the retina can resist this insult because of their elasticity but Bruch's membrane breaks because it does not have enough tensile strength. Small capillaries in the choriocapillaris and deep choroidal vessels are damaged leading to sub-RPE hemorrhages.

Choroidal neovascularization (CNV) is a common complication associated with traumatic choroidal rupture [2]. Bevacizumab, a humanized monoclonal antibody to vascular endothelial growth factor, has been given as an intravitreal injection to treat choroidal neovascularization (CNV) secondary to CR [3]. We used a non-invasive imaging modality, optical coherence tomography angiography (OCTA), to detect and follow the progression of the CNV in a patient with traumatic choroidal rupture.

## 2. Case Report

**Figure 1:**
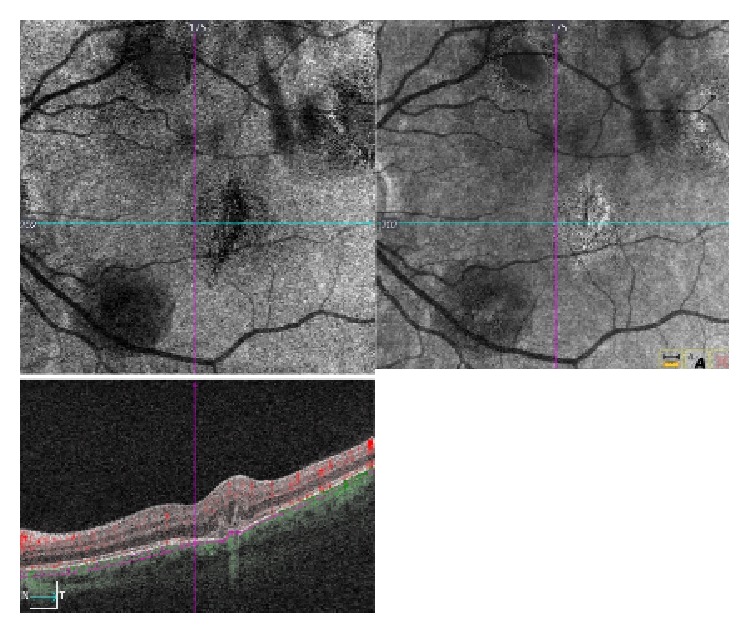
**Baseline examination of choroidal rupture. **OCTA shows a hyporeflective line corresponding to retinal pigment epithelium (RPE)-choriocapillaris defects near to the foveal area. B-scan reveals a disruption of the RPE-Bruch membrane and a hyperreflective intraretinal area associated with backscattering effect was also present.

**Figure 2:**
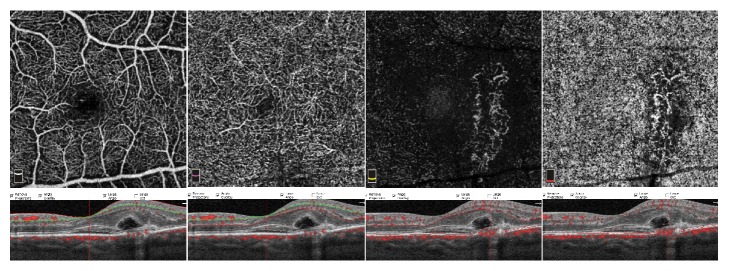
**Optical coherence tomography angiography (OCTA): enface angiograms at 4-week visit. **Angiograms of outer retina (A) and choriocapillaris (B) demonstrate the presence of a well circumscribed lesion with a clear hyperintense signal and characterized by numerous anastomotic vessels. Neovascular lesion extends along the primary RPE-Bruch membrane defect. OCT B-scan shows subretinal fluid.

**Figure 3:**
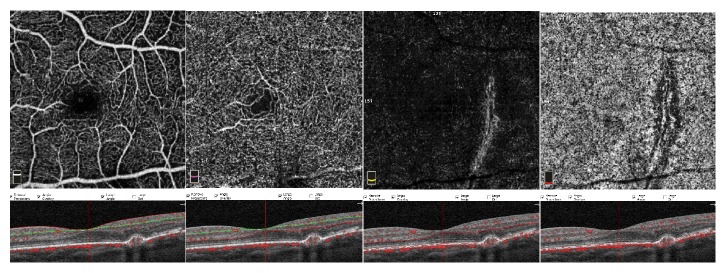
**Optical coherence tomography angiography (OCTA): enface angiograms at 6-week visit. **OCTA reveals shrinkage of the choriocapillaris defect; enface scan of outer retina and choriocapillaris show no hyperintense flow signal. OCT B-scan demonstrates partial healing of the RPE wound, covered by a less reflective RPE and a reduction of hyperreflective intraretinal material; no subretinal fluid is evident.

A healthy 25-year-old man presented with recent visual acuity reduction in the left eye (LE) after a blunt ocular trauma he received the day before. Complete ophthalmologic evaluation showed best-corrected visual acuity (BCVA) of 20/20 in the RE and of 20/25 in the LE. Slit-lamp biomicroscopy examination of the anterior segment of the LE showed conjunctival hyperemia and normal pupillary reflex to light. The anterior segment of the RE was normal. Intraocular pressure was 17 mmHg in both eyes. Ophthalmoscopic examination of the LE revealed commotio retinae in the inferior quadrants, four subretinal macular hemorrhages, and two choroidal ruptures located temporally to the fovea. Fundus examination of the RE was unremarkable. The patient underwent imaging with fluorescein angiography (FA), spectral domain optical coherence tomography (SD-OCT), and optical coherence tomography angiography (OCT-A). On FA, choroidal ruptures (CR) were hyperfluorescent in the mid and late phases due to staining. Hemorrhages appeared as round hypofluorescent areas. SD-OCT scans revealed disruption of the retinal pigment epithelium (RPE)-Bruch membrane (BM) complex associated with back-scattering effect. OCTA allows a depth resolved visualization of retinal and choriocapillaris microvasculature. On OCT angiograms, the choroidal rupture appeared as a hypointense break in choriocapillaris plexus (Figure 1). At 4-week follow-up, FA was repeated and revealed leakage in the perifoveal area in correspondence of the CR. SD-OCT showed an hyperreflective lesion involving the ellipsoid zone (EZ) and outer nuclear layer such as hyporeflective subretinal fluid present next to the choroidal rupture. In the OCT-A enface scan, taken above the retinal pigment epithelium, a well circumscribed lesion with a clear hyperintense signal was observed. Lesion was characterized by numerous and fine anastomotic vessels with a well-shaped peripheral arcade (Figure 2). Superficial and deep retinal vasculature were spared. Diagnosis of choroidal neovascular membrane (CNV) secondary to choroidal rupture was done. After a written consent was signed by the patient, an off-label intravitreal injection of Bevacizumab (1.25 mg/0.05 ml) was administered in his LE. At 6-week post injection, visual acuity has improved to 20/20. OCTA documented the closure of CNV (Figure 3). C-scans of the outer retina and choriocapillaris did not show any hyperintense signal. SD-OCT revealed RPE wound healing process as lesion covered by a less hyperreflective layer of RPE. This layer was less hyperreflective than normal RPE.

## 3. Discussion

Choroidal ruptures are breaks in the retinal pigment epithelium, Bruch's membrane, and choroid that occur in 5% of blunt ocular trauma [4]. CR may be complicated with haemorrhagic or serous macular detachment, optic disc pallor, and choroidal neovascularization [1]. The formation of CNV must consider the distance of the CR from the fovea and the length of the break [2].

In this report, we used OCT-A, a new imaging tool, to investigate the choroidal and retinal vasculature in a patient with traumatic choroidal rupture. OCT-A is a recent, non-invasive imaging technology, able to visualize the microvasculature without any dye injection [5]. OCT-A shows an in vivo three-dimensional vasculature of the retina and choroid and performs a segmentation in different layers. We used OCT-A initially to evaluate and describe the vascular structure of the area near the traumatic choroidal rupture. Monitoring the patient with OCT-A, we observed the presence of CNV and its regression after anti-VEGF therapy.

Choroidal neovascularization is a well-known complication of traumatic choroidal rupture. Most of the post-traumatic membranes are sub- or juxta-foveal [6]. The more abundant blood supply from the choroidal vasculature and the more intense healing response may be linked to the increased propensity to CNV in the fovea [7]. Many growth factors, including those of the VEGF family, are involved in wound healing process. A local higher level of elastin-derived peptides released from the rupture of Bruch membrane could induce VEGF upregulation [8]. Anti-VEGF therapy is recognized as gold standard treatment of post-traumatic CNV [9].

In clinical practice, OCT-A represents a new non-invasive method that provides important information about vascular flow in macular area and represents an early diagnostic tool for CNV detection that can also be used to follow its progression over time.

To our knowledge, for the first time, we used OCT angiography to early detect and follow the evolution of CNV, one of the common complications of choroidal rupture. OCT-A is a fast, non-invasive imaging tool that can be used for an early diagnosis of disease. This imaging technology might provide relevant information about microvascular response to anti-VEGF therapy and wound healing process without use of exogenous dye. Further studies are needed to establish the value of OCT-A in early diagnosis of CNV in blunt ocular trauma with choroidal rupture and to assess the role in the follow-up of the lesion.

## Additional Points

*Summary*. The authors report a case of a 25-year-old man with choroidal neovascularization (CNV) secondary to traumatic choroidal rupture. OCT angiography was used to evaluate the vascular structure of the area near the traumatic choroidal rupture at baseline visit and during follow-up.
